# 2,6,6-Trimethyl­cyclo­hexene-1-carbaldehyde oxime

**DOI:** 10.1107/S1600536811037895

**Published:** 2011-09-30

**Authors:** Rajasekaran Parthasarathy, Samson Jegan Jenniefer, Packianathan Thomas Muthiah, Nagarajan Sulochana

**Affiliations:** a Department of Chemistry, National Institute of Technology, Tiruchirappalli 620 015, India; b School of Chemistry, Bharathidasan University, Tiruchirappalli 620 024, Tamilnadu, India; c Department of Chemistry, National Institute of Technology, Karaikal 609 605, India

## Abstract

In the crystal of the title compound C_10_H_17_NO, synthesized by the reaction of β-cyclo­citral with hydroxyl­amine hydro­chloride, inversion-related mol­ecules are linked by a pair of O—H⋯N hydrogen-bonding inter­actions between the oxime functionalities, forming *R*_2_^2^(6) loops. The molecular conformation is stabilized by intra­molecular methyl C—H⋯N inter­actions. The cyclohexene ring has the typical half-chair conformation.

## Related literature

For applications of oximes in organic syntheses, see: Cerny *et al.* (1969 ▶); Donaruma & Heldt (1960 ▶); Kutney *et al.* (1992 ▶); Touster (1953 ▶). For graph-set notation, see: Etter *et al.* (1990 ▶); Bernstein *et al.* (1995 ▶).
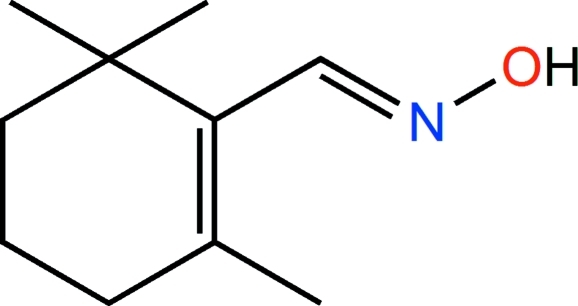


## Experimental

### 

#### Crystal data

C_10_H_17_NO
*M* *_r_* = 167.25
Triclinic, 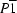

*a* = 7.5670 (3) Å
*b* = 7.7208 (3) Å
*c* = 9.3072 (4) Å
α = 81.212 (3)°
β = 76.590 (3)°
γ = 71.385 (3)°
*V* = 499.43 (4) Å^3^
*Z* = 2
Mo *K*α radiation
μ = 0.07 mm^−1^
*T* = 296 K
0.09 × 0.06 × 0.05 mm

#### Data collection

Bruker SMART APEXII CCD area-detector diffractometer
Absorption correction: multi-scan (*SADABS*; Bruker, 2008 ▶) *T*_min_ = 0.994, *T*_max_ = 0.997
13971 measured reflections
3341 independent reflections
2134 reflections with *I* > 2σ(*I*)
*R*_int_ = 0.022

#### Refinement

*R*[*F* ^2^ > 2σ(*F* ^2^)] = 0.053
*wR*(*F* ^2^) = 0.191
*S* = 1.06
3341 reflections
118 parameters
H atoms treated by a mixture of independent and constrained refinement
Δρ_max_ = 0.24 e Å^−3^
Δρ_min_ = −0.14 e Å^−3^

### 

Data collection: *APEX2* (Bruker, 2008 ▶); cell refinement: *SAINT* (Bruker, 2008 ▶); data reduction: *SAINT*; program(s) used to solve structure: *SHELXS97* (Sheldrick, 2008 ▶); program(s) used to refine structure: *SHELXL97* (Sheldrick, 2008 ▶); molecular graphics: *PLATON* (Spek, 2009 ▶); software used to prepare material for publication: *PLATON*.

## Supplementary Material

Crystal structure: contains datablock(s) global, I. DOI: 10.1107/S1600536811037895/zs2144sup1.cif

Structure factors: contains datablock(s) I. DOI: 10.1107/S1600536811037895/zs2144Isup2.hkl

Supplementary material file. DOI: 10.1107/S1600536811037895/zs2144Isup3.cml

Additional supplementary materials:  crystallographic information; 3D view; checkCIF report

## Figures and Tables

**Table 1:** Hydrogen-bond geometry (Å, °)

*D*—H⋯*A*	*D*—H	H⋯*A*	*D*⋯*A*	*D*—H⋯*A*
O1—H1⋯N1^i^	0.86 (3)	2.02 (3)	2.8346 (18)	158 (2)
C9—H9*A*⋯N1	0.96	2.57	3.1979 (19)	123
C10—H10*A*⋯N1	0.96	2.43	3.0762 (17)	125

Symmetry code: (i) 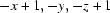.

**Fig. 1.**
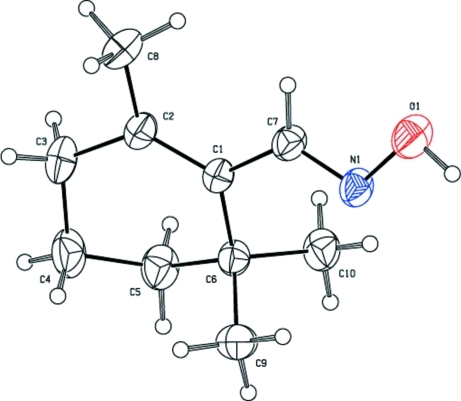
The molecular structure of the title compound, with atom labels and anisotropic displacement ellipsoids drawn at the 50% probability level.

**Fig. 2.**
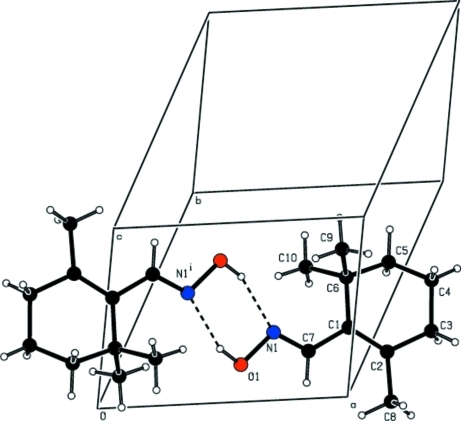
The centrosymmetric R22(6) hydrogen-bonded dimer units, with hydrogen bonds shown as dashed lines. For symmetry code (i), see Table 1.

## supplementary crystallographic information

### Comment

An oxime is an important functional group in organic chemistry because it is not only used as an efficient protecting group for carbonyls but also may be used for the purification of carbonyl compounds (Donaruma & Heldt, 1960). Moreover oximes are used for the preparation of many compounds such as amines by reduction (Cerny *et al.*, 1969), nitro compounds by oxidation, amides by the Beckmann rearrangement and carbonyl compounds from non carbonyl compounds (Touster, 1953). The title compound C_10_H_17_NO is a key intermediate in the synthesis of aroma compounds such as β-cyclogeranyl nitrile which can be used for the synthesis of the important aroma compound β-damascone (Kutney *et al.*, 1992). Herein, we report the crystal structure of the title compound (Fig. 1) in which each molecule is connected to an inversion-related molecule through O—H···N hydrogen bonds, (Table 1) forming a cyclic dimer [graph-set *R*^2^_2_(6) (Etter *et al.*, 1990; Bernstein *et al.*, 1995] (Fig. 2). These cyclic DA—AD (Donor Acceptor–Acceptor Donor) interactions involving pairs of O—H···N hydrogen bonds between the oxime functionalities are similar to the O—H···O interactions observed in carboxylic acid dimers. The crystal structure is stabilized by intramolecular methyl C—H···N_oxime_ hydrogen-bonding interactions.

### Experimental

To a mixture of 4.6 g (0.065 mol) of hydroxylamine hydrochloride in 50 ml of H_2_O and 10 g (0.065 mol) of β-cyclocitral, a solution of 3.5 g (0.033 mol) of sodium carbonate in 15 ml of H_2_O was added dropwise. The mixture was stirred at room temperature for ten minutes and the solid product which formed was collected and recrystallized from hexane.

### Refinement

The H atoms attached to C7 and O1 were located from a difference Fourier map and were refined freely. The remaining H atoms were placed in geometrically idealized positions and constrained to ride on their parent atoms, with C—H distances in the range 0.93–0.97 Å, and with *U*_iso_(H) set at 1.2*U*_eq_(C) except for the methyl hydrogen atoms which were refined with *U*_iso_(H) set at 1.5*U*_eq_(C).

### Crystal data

**Table:**

C_10_H_17_NO	*Z* = 2
*M**_r_* = 167.25	*F*(000) = 184
Triclinic, *P*1	*D*_x_ = 1.112 Mg m^−^^3^
Hall symbol: -P 1	Mo *K*α radiation, λ = 0.71073 Å
*a* = 7.5670 (3) Å	Cell parameters from 3341 reflections
*b* = 7.7208 (3) Å	θ = 2.3–33.0°
*c* = 9.3072 (4) Å	µ = 0.07 mm^−^^1^
α = 81.212 (3)°	*T* = 296 K
β = 76.590 (3)°	Prism, colourless
γ = 71.385 (3)°	0.09 × 0.06 × 0.05 mm
*V* = 499.43 (4) Å^3^	

### Data collection

**Table:**

Bruker SMART APEXII CCD area-detector diffractometer	3341 independent reflections
Radiation source: fine-focus sealed tube	2134 reflections with *I* > 2σ(*I*)
graphite	*R*_int_ = 0.022
φ and ω scans	θ_max_ = 33.0°, θ_min_ = 2.3°
Absorption correction: multi-scan (*SADABS*; Bruker, 2008)	*h* = −11→11
*T*_min_ = 0.994, *T*_max_ = 0.997	*k* = −11→11
13971 measured reflections	*l* = −12→13

### Refinement

**Table:**

Refinement on *F*^2^	Primary atom site location: structure-invariant direct methods
Least-squares matrix: full	Secondary atom site location: difference Fourier map
*R*[*F*^2^ > 2σ(*F*^2^)] = 0.053	Hydrogen site location: inferred from neighbouring sites
*wR*(*F*^2^) = 0.191	H atoms treated by a mixture of independent and constrained refinement
*S* = 1.06	*w* = 1/[σ^2^(*F*_o_^2^) + (0.0999*P*)^2^ + 0.036*P*] where *P* = (*F*_o_^2^ + 2*F*_c_^2^)/3
3341 reflections	(Δ/σ)_max_ < 0.001
118 parameters	Δρ_max_ = 0.24 e Å^−^^3^
0 restraints	Δρ_min_ = −0.14 e Å^−^^3^

### Special details

**Table:**

Geometry. Bond distances, angles *etc*. have been calculated using the rounded fractional coordinates. All su's are estimated from the variances of the (full) variance-covariance matrix. The cell e.s.d.'s are taken into account in the estimation of distances, angles and torsion angles
Refinement. Refinement on *F*^2^ for ALL reflections except those flagged by the user for potential systematic errors. Weighted *R*-factors *wR* and all goodnesses of fit *S* are based on *F*^2^, conventional *R*-factors *R* are based on *F*, with *F* set to zero for negative *F*^2^. The observed criterion of *F*^2^ > σ(*F*^2^) is used only for calculating -*R*-factor-obs *etc*. and is not relevant to the choice of reflections for refinement. *R*-factors based on *F*^2^ are statistically about twice as large as those based on *F*, and *R*-factors based on ALL data will be even larger.

### Fractional atomic coordinates and isotropic or equivalent isotropic displacement parameters (Å^2^)

**Table:**

	*x*	*y*	*z*	*U*_iso_*/*U*_eq_	
O1	0.60371 (16)	−0.14350 (15)	0.37650 (16)	0.0794 (5)	
N1	0.67895 (14)	0.00174 (14)	0.37695 (13)	0.0552 (4)	
C1	0.94463 (15)	0.11492 (15)	0.25083 (13)	0.0425 (3)	
C2	1.12581 (16)	0.05523 (17)	0.17581 (14)	0.0492 (3)	
C3	1.26022 (18)	0.1703 (2)	0.1444 (2)	0.0680 (5)	
C4	1.1923 (2)	0.3358 (2)	0.2335 (2)	0.0772 (6)	
C5	0.9848 (2)	0.42925 (19)	0.2342 (2)	0.0656 (5)	
C6	0.85762 (15)	0.30698 (15)	0.30624 (14)	0.0458 (3)	
C7	0.83019 (17)	−0.01188 (16)	0.27932 (16)	0.0505 (4)	
C8	1.2169 (2)	−0.1322 (2)	0.11773 (18)	0.0689 (5)	
C9	0.8338 (2)	0.2967 (2)	0.47500 (17)	0.0641 (5)	
C10	0.66297 (19)	0.40088 (18)	0.26268 (19)	0.0627 (5)	
H1	0.509 (3)	−0.126 (3)	0.450 (3)	0.107 (7)*	
H3A	1.27880	0.21180	0.03980	0.0820*	
H3B	1.38250	0.09430	0.16530	0.0820*	
H4A	1.21150	0.29760	0.33440	0.0930*	
H4B	1.26500	0.42070	0.19030	0.0930*	
H5A	0.96760	0.46760	0.13290	0.0790*	
H5B	0.94450	0.53850	0.28720	0.0790*	
H7	0.866 (2)	−0.116 (2)	0.2260 (19)	0.074 (5)*	
H8A	1.29070	−0.21220	0.18590	0.1030*	
H8B	1.29810	−0.12140	0.02290	0.1030*	
H8C	1.11960	−0.18180	0.10750	0.1030*	
H9A	0.75450	0.22010	0.52080	0.0960*	
H9B	0.77580	0.41760	0.50780	0.0960*	
H9C	0.95610	0.24580	0.50220	0.0960*	
H10A	0.57850	0.32850	0.30550	0.0940*	
H10B	0.67810	0.41230	0.15670	0.0940*	
H10C	0.61080	0.52050	0.29870	0.0940*	

### Atomic displacement parameters (Å^2^)

**Table:**

	*U*^11^	*U*^22^	*U*^33^	*U*^12^	*U*^13^	*U*^23^
O1	0.0698 (7)	0.0627 (6)	0.1080 (10)	−0.0392 (5)	0.0201 (6)	−0.0293 (6)
N1	0.0466 (5)	0.0490 (5)	0.0702 (8)	−0.0218 (4)	0.0031 (5)	−0.0113 (5)
C1	0.0377 (5)	0.0448 (5)	0.0424 (6)	−0.0118 (4)	−0.0047 (4)	−0.0018 (4)
C2	0.0404 (5)	0.0555 (6)	0.0453 (7)	−0.0109 (5)	−0.0021 (5)	−0.0023 (5)
C3	0.0404 (6)	0.0793 (9)	0.0796 (11)	−0.0225 (6)	0.0014 (6)	−0.0022 (8)
C4	0.0539 (8)	0.0782 (10)	0.1078 (14)	−0.0362 (7)	−0.0069 (8)	−0.0105 (9)
C5	0.0586 (8)	0.0526 (7)	0.0853 (11)	−0.0243 (6)	−0.0051 (7)	−0.0019 (7)
C6	0.0399 (5)	0.0433 (5)	0.0530 (7)	−0.0131 (4)	−0.0055 (5)	−0.0046 (5)
C7	0.0459 (6)	0.0439 (6)	0.0588 (8)	−0.0136 (4)	0.0000 (5)	−0.0105 (5)
C8	0.0548 (7)	0.0692 (9)	0.0673 (10)	−0.0053 (6)	0.0068 (7)	−0.0172 (7)
C9	0.0694 (8)	0.0688 (8)	0.0574 (8)	−0.0237 (7)	−0.0063 (7)	−0.0171 (7)
C10	0.0491 (7)	0.0496 (7)	0.0859 (11)	−0.0041 (5)	−0.0184 (7)	−0.0100 (6)

### Geometric parameters (Å, °)

**Table:**

O1—N1	1.4113 (16)	C4—H4A	0.9700
O1—H1	0.86 (3)	C4—H4B	0.9700
N1—C7	1.2714 (18)	C5—H5A	0.9700
C1—C6	1.5334 (16)	C5—H5B	0.9700
C1—C7	1.4608 (18)	C7—H7	0.942 (15)
C1—C2	1.3530 (18)	C8—H8A	0.9600
C2—C8	1.5136 (19)	C8—H8B	0.9600
C2—C3	1.506 (2)	C8—H8C	0.9600
C3—C4	1.514 (2)	C9—H9A	0.9600
C4—C5	1.503 (2)	C9—H9B	0.9600
C5—C6	1.534 (2)	C9—H9C	0.9600
C6—C9	1.532 (2)	C10—H10A	0.9600
C6—C10	1.538 (2)	C10—H10B	0.9600
C3—H3A	0.9700	C10—H10C	0.9600
C3—H3B	0.9700		
			
N1—O1—H1	103.3 (15)	H4A—C4—H4B	108.00
O1—N1—C7	111.09 (11)	C4—C5—H5A	109.00
C2—C1—C6	122.81 (11)	C4—C5—H5B	109.00
C6—C1—C7	119.71 (10)	C6—C5—H5A	109.00
C2—C1—C7	117.48 (11)	C6—C5—H5B	109.00
C1—C2—C8	124.63 (12)	H5A—C5—H5B	108.00
C3—C2—C8	112.81 (12)	N1—C7—H7	113.3 (10)
C1—C2—C3	122.55 (12)	C1—C7—H7	121.4 (10)
C2—C3—C4	113.94 (13)	C2—C8—H8A	109.00
C3—C4—C5	109.66 (13)	C2—C8—H8B	109.00
C4—C5—C6	113.33 (12)	C2—C8—H8C	109.00
C1—C6—C9	110.71 (10)	H8A—C8—H8B	109.00
C1—C6—C10	110.80 (10)	H8A—C8—H8C	109.00
C5—C6—C9	109.05 (12)	H8B—C8—H8C	109.00
C5—C6—C10	106.66 (11)	C6—C9—H9A	109.00
C9—C6—C10	109.18 (11)	C6—C9—H9B	109.00
C1—C6—C5	110.33 (10)	C6—C9—H9C	109.00
N1—C7—C1	125.29 (12)	H9A—C9—H9B	109.00
C2—C3—H3A	109.00	H9A—C9—H9C	109.00
C2—C3—H3B	109.00	H9B—C9—H9C	110.00
C4—C3—H3A	109.00	C6—C10—H10A	109.00
C4—C3—H3B	109.00	C6—C10—H10B	109.00
H3A—C3—H3B	108.00	C6—C10—H10C	109.00
C3—C4—H4A	110.00	H10A—C10—H10B	110.00
C3—C4—H4B	110.00	H10A—C10—H10C	109.00
C5—C4—H4A	110.00	H10B—C10—H10C	109.00
C5—C4—H4B	110.00		
			
O1—N1—C7—C1	−179.61 (12)	C7—C1—C6—C10	−49.10 (16)
C6—C1—C2—C3	2.0 (2)	C2—C1—C7—N1	161.31 (13)
C6—C1—C2—C8	−179.34 (12)	C6—C1—C7—N1	−18.5 (2)
C7—C1—C2—C3	−177.72 (13)	C1—C2—C3—C4	13.8 (2)
C7—C1—C2—C8	0.89 (19)	C8—C2—C3—C4	−165.00 (13)
C2—C1—C6—C5	13.24 (17)	C2—C3—C4—C5	−44.02 (19)
C2—C1—C6—C9	−107.58 (14)	C3—C4—C5—C6	61.57 (18)
C2—C1—C6—C10	131.13 (13)	C4—C5—C6—C1	−45.18 (17)
C7—C1—C6—C5	−167.00 (12)	C4—C5—C6—C9	76.62 (16)
C7—C1—C6—C9	72.19 (15)	C4—C5—C6—C10	−165.59 (13)

### Hydrogen-bond geometry (Å, °)

**Table:**

*D*—H···*A*	*D*—H	H···*A*	*D*···*A*	*D*—H···*A*
O1—H1···N1^i^	0.86 (3)	2.02 (3)	2.8346 (18)	158 (2)
C9—H9A···N1	0.96	2.57	3.1979 (19)	123
C10—H10A···N1	0.96	2.43	3.0762 (17)	125

Symmetry codes: (i) −*x*+1, −*y*, −*z*+1.
